# Vocal Cord Paralysis and its Etiologies: A Prospective Study

**DOI:** 10.5681/jcvtr.2014.009

**Published:** 2014-03-04

**Authors:** Seyed Javad Seyed Toutounchi, Mahmood Eydi, Samad EJ Golzari, Mohammad Reza Ghaffari, Nashmil Parvizian

**Affiliations:** ^1^Department of ENT, Tabriz University of Medical Sciences, Tabriz, Iran; ^2^Department of Anesthesiology, Tabriz University of Medical Sciences, Tabriz, Iran; ^3^Cardiovascular Research Center, Tabriz University of Medical Sciences, Tabriz, Iran

**Keywords:** Vocal Cord, Larynx, Paralysis, Etiology

## Abstract

***Introduction:*** Vocal cord paralysis is a common symptom of numerous diseases and it may be due to neurogenic or mechanical fixation of the cords. Paralysis of the vocal cords is just a symptom of underlying disease in some cases; so, clinical diagnosis of the underlying cause leading to paralysis of the vocal cords is important. This study evaluates the causes of vocal cord paralysis.

***Methods:*** In a prospective study, 45 patients with paralyzed vocal cord diagnosis were examined by tests such as examination of the pharynx, larynx, esophagus, thyroid, cervical, lung, and mediastinum, brain and heart by diagnostic imaging to investigate the cause vocal cord paralysis. The study was ended by diagnosing the reason of vocal cord paralysis at each stage of the examination and the clinical studies.

***Results:*** The mean duration of symptoms was 18.95±6.50 months. The reason for referral was phonation changes (97.8%) and aspiration (37.8%) in the subjects. There was bilateral paralysis in 6.82%, left paralysis in 56.82% and right in 63.36% of subjects. The type of vocal cord placement was midline in 52.8%, paramedian in 44.4% and lateral in 2.8% of the subjects. The causes of vocal cords paralysis were idiopathic paralysis (31.11%), tumors (31.11%), surgery (28.89%), trauma, brain problems, systemic disease and other causes (2.2%).

***Conclusion:*** An integrated diagnostic and treatment program is necessary for patients with vocal cord paralysis. Possibility of malignancy should be excluded before marking idiopathic reason to vocal cord paralysis.

## Introduction

Management of the airway has always been of great importance throughout the history^1^ and ancient physician have focused on the disease contributing to the airway and respiratory system.^2-5^ Vocal cord paralysis is a common symptom of the disease which can be originated from laryngeal nerve paralysis following surgical procedures^6,7^, post-anesthesia complication^8-10^ or neurologic diseases.^11-14^ Laryngeal nerve paralysis of the abductors often leads to para-median positioning of vocal cords. Symptoms include hoarseness, dysphonia, dyspnea and aspiration.^15-17^ Failure in the movement of the vocal cords can also be due to mechanical fixation**.**^18^**** Paralysis of the vocal cords is just a symptom of underlying disease in some cases.^19^ Diagnosing initial disease is remarkable that in case of late diagnosis could result in serious symptoms or death such as malignant cancers.^19,20^ Recurrent laryngeal nerve function can be impaired due to pressure or pathology of the disease damaging the nerve leading to paralysis of the vocal cords.^21^ Malignant invasion to vagus nerve or recurrent laryngeal nerve invasion of malignant neoplasms can be generated by the thyroid neoplasms, lung cancer, esophagus carcinoma and mediastinal metastasis.^22,23^ Initial studies found that tumors are the common cause of cancer; bronchogenic carcinoma is the most usual cause of unilateral paralysis of vocal cords.^23-26^ Left laryngeal nerve is more vulnerable than the right because travels a longer route in thoracic cavity is placed in the proximity of the left lobe of lung; then, it continues its route toward mediastinal lymph nodes and eventually loops around the aortic arch.^21^ Paralysis of the left vocal cord is reported 1.4-2.5 time more than right.^20^ Endotracheal intubation carried out for elective surgical procedures can lead to pathological changes, trauma and nerve damage. It seems that the pressure of cuff is related with nerve paralysis caused by nerve pressure and neuropraxia.^26^ However, there are few reports of bilateral vocal cord paralysis caused by endotracheal tubes.^27^ Idiopathic and viral paralysis routinely heals before they need medical intervention. The patients assessment with vocal cord paralysis include complete description, a full evaluation of the head and neck, larynx and neurological, chest X-ray and CT scan or MRI of the skull base to the thoracic inlet for eliminating the brain stem, neck, chest and mediastinum causes.^28,29^

Yumoto et al. study between 1987 and 1997 as well as the study of Rosenthal et al. demonstrated Surgery and idiopathic factors as the main cause of unilateral vocal cord paralysis.^30,31^ The overall aim of our study was to determine the prevalence of various factors causing paralysis of the vocal cords by considering changing incidence of cancer, virulence of infectious agents, new surgical procedures especially heart surgery and anesthesia procedures such as new endotracheal tube and cuff design.

## Materials and methods

In a cross-sectional study, 45 subjects with hoarseness or aspiration following consultation from various departments or directly referred to the Imam Reza Hospital participated in the study after diagnosis of vocal cord paralysis by laryngoscopy with a 90° telescope was confirmed. Subject sampling was performed in a census from July 2010 to July 2011. All patients underwent further investigations such as full endoscopic examination of the larynx, pharynx, esophagus, thyroid, neck, lung and mediastinum, brain and heart using diagnostic procedures such as CT scans, MRI, barium swallow and thyroid scan. By revealing the cause of vocal cord paralysis at each stage of clinical examinations and paraclinical evaluations procedure stopped. All variables such as age, sex, duration of paralysis, clinical signs, laryngoscopic findings, unilateral or bilateral paralysis, diagnostic tests and history of brain, chest, heart, thyroid, esophagus and larynx surgery were collected; all patients’ information was kept confidential. All data were analyzed using SPSS 16. Descriptive statistics (frequency, percentage) were used for statistical analysis. Chi-square test was used for comparison of quantitative variables. The quantitative comparison between the three groups was accomplished by one-Way ANOVA test; P value of less than 0.05 in this study was considered significant.

## Results

**Figure 1:**
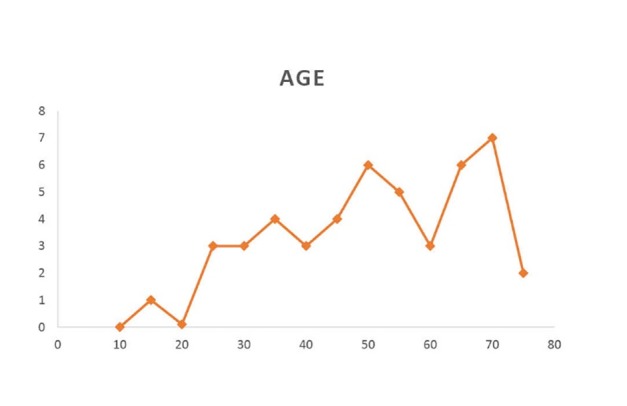
Distribution of age

**Figure 2:**
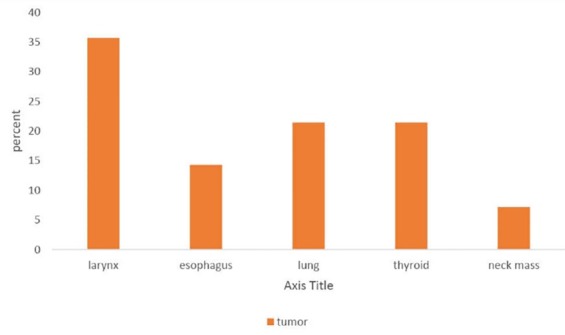
Frequency of various malignancies

**Figure 3:**
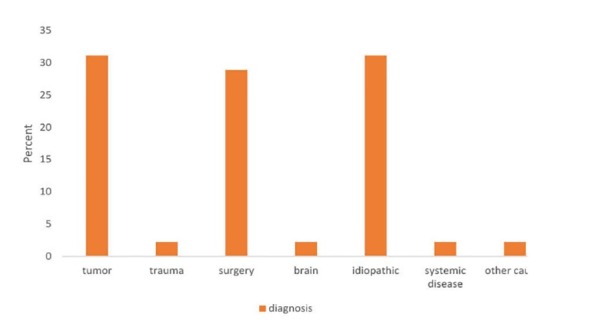
The final diagnosis of vocal cord paralysis

**Figure 4:**
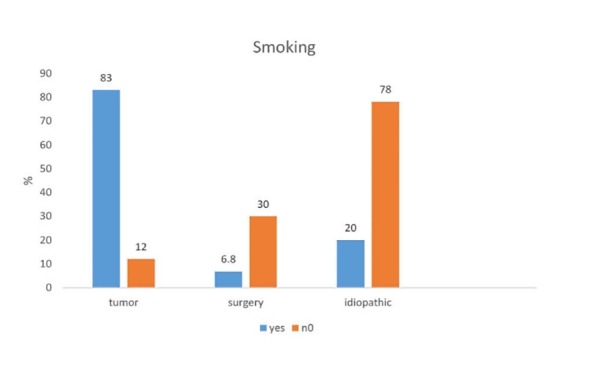
Prevalence of smoking among common cause of vocal cord paralysis

Totally 45 patients diagnosed with vocal cord paralysis were studied. Among subjects, 31 patients (68.9%) were male and 14 (31.1%) were female. The median and mode age of patients was 53 and 37 years old. The youngest and oldest patients were 14 and 74 years respectively (Figures 1-4). The mean of disease duration was 18.95±6.50 months with median of 4 months. Two subjects had history of vocal cords paralysis for more than a year. Smoking history was positive in 27 patients (60%). The most common symptoms were phonation change (97.8%) and aspiration (37.8%). Paralysis after was observed in 14 patients (31.1%) after general surgery, 9 patient (20%) after cardiac surgery, 4 patients (8.8%) after thyroid surgery cases, 1 patient (2.2%) in brain surgery which subjects were under general anesthesia and endotracheal intubation. There were 9 cases (20%) with ICU history and 1 head trauma case. In 14 patients (31.1%) tumor was most frequent cause of vocal cord paralysis with laryngeal cancer being the most common between them (Figures 2-4). There were 2 patients with a history of cancer radiotherapy and 1 with chemotherapy history.

**Figure 5:**
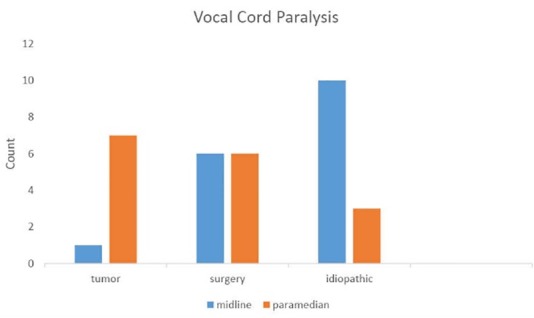
Location of vocal cord paralysis in common

The left-sided involvement was the most frequent type (56.82%); right sided was 36.36% and bilateral paralysis 6.8%. In 52.8% cases, vocal cord was at the midline position, 44.4% paramedian and 2.8% lateral. CT findings in 14 patients, thyroid scan in 6 cases of, MRI in 3 cases and barium swallow in 1 patient were abnormal. Figures 3-4 show final diagnosis in patients with vocal cord paralysis. It was observed that a majority of patients have vocal cord paralysis because of tumor or surgery. Three causes of vocal cord paralysis include surgery, tumor and idiopathic were compared separately. Figure 4 shows prevalence of smoking among 3 common causes of vocal cord paralysis. As it can be seen, smoking was significantly higher in tumor and surgery than idiopathic cases (P=0.002). There was aspiration in 7 cases (50%) following paralysis caused by tumor, in 5 cases (38.8%) cases were associated with surgery and 3 (21.4%) of cases were idiopathic. No significant difference was found between the three groups in terms of aspiration (P=0.28). There was no significant difference between affected side, unilateral and bilateral paralysis of the three groups. The duration of symptoms was lowest in subjects with tumor, but no significant difference was found between the three groups (P=0.5). Figures 4-5 show the locations of the vocal cords in three common factors which were paramedian and midline in in tumor and idiopathic cases, respectively (P=0.01). In this study, the most common causes of vocal cord paralysis in men and in women were tumors (35.5%) and idiopathic (50%), respectively.

## Discussion

The symptoms of vocal cord paralysis include changes in voice, aspiration and respiratory problems that are the same in all studies.^15-17^ Paralysis of the left vocal cord has been reported 1.4-2.5 times more than right side.^20^ In our study, bilateral paralysis was 6.82%, left paralysis 56.82% and right 36.36% that was consistent with Ko et al. reporting nearly 68% paralysis in left side^32^ and Srirompotong et al. reporting 73% of the paralysis in left side.^33^ Numerous studies have explained four major causes for unilateral vocal cord paralysis including surgery, malignancy, idiopathic and neck trauma. Ramadan et al., in their study between 1991 and 1994, found that malignancy in 31.6%, Surgery in 29.6%, idiopathic in 16.3% and neck trauma in 7.1% of patients were the causes of paralysis.^34^

Yumoto et al. reported surgery in 42.7%, malignancy in 22.4%, idiopathic in 17.4% and injuries of the neck in 2.2% of cases as unilateral paralysis vocal cord etiology.^30^ Rosenthal et al. stated surgery in 46.3%, malignancy in 13.5%, idiopathic in 17.6% and neck trauma in 2.2% of subjects as reason of unilateral vocal cord paralysis.^31^ In our study, idiopathic in 31.11%, tumor in 31.11%, Surgery in 28.89% and trauma, brain problems, systemic disease and other causes each in 2.2% of cases were causes of vocal cord disease.

Chen et al. reported lung cancer as the etiology for paralysis in 34 cases.^23^ Ko et al. found that thyroid and lung tumors were the most common sites of tumor origin.^32^ However, the most common tumors in our study were larynx, thyroid lung and tumors, respectively.

In Chen et al. and Ko et al. studies, thyroidectomy was the most common reason of surgery for vocal cord paralysis.^23,32^ Unlike findings in our study, the most popular causes of paralysis following surgery were heart and then thyroid surgeries, respectively. Chen et al. found malignancy in males; while, the most common cause for vocal cord paralysis in females was surgery.^23^ In the present study, tumors in paramedian position were the most frequent etiological factor for vocal fold paralysis in men; while, idiopathic cases constitute 50% of cases in women in midline position. Considering the position of the paralyzed vocal cord throughout the diagnosis and laryngoscopy could be helpful and time-saving.

## Conclusion

An integrated diagnostic and treatment program is necessary for patients with vocal fold paralysis as because various factors are responsible for the disease. Prior to marking as idiopathic vocal cord paralysis, probability of cancer should be reviewed and eliminated.

## Ethical issues

All patients gave written informed consents and the study was approved by our local Ethics Committee.

## Competing interests

The authors declare that they have no competing interests.
